# Variations in the Post-weaning Human Gut Metagenome Profile As Result of *Bifidobacterium* Acquisition in the Western Microbiome

**DOI:** 10.3389/fmicb.2016.01058

**Published:** 2016-07-12

**Authors:** Matteo Soverini, Simone Rampelli, Silvia Turroni, Stephanie L. Schnorr, Sara Quercia, Andrea Castagnetti, Elena Biagi, Patrizia Brigidi, Marco Candela

**Affiliations:** ^1^Department of Pharmacy and Biotechnology, University of BolognaBologna, Italy; ^2^Department of Anthropology, University of OklahomaNorman, OK, USA

**Keywords:** *Bifidobacterium*, *Treponema*, gut microbiota, microbiome, co-evolution, Westernization

## Abstract

Studies of the gut microbiome variation among human populations revealed the existence of robust compositional and functional layouts matching the three subsistence strategies that describe a trajectory of changes across our recent evolutionary history: hunting and gathering, rural agriculture, and urban post-industrialized agriculture. In particular, beside the overall reduction of ecosystem diversity, the gut microbiome of Western industrial populations is typically characterized by the loss of *Treponema* and the acquisition of *Bifidobacterium* as an abundant inhabitant of the post-weaning gut microbial ecosystem. In order to advance the hypothesis about the possible adaptive nature of this exchange, here we explore specific functional attributes that correspond to the mutually exclusive presence of *Treponema* and *Bifidobacterium* using publically available gut metagenomic data from Hadza hunter-gatherers and urban industrial Italians. According to our findings, *Bifidobacterium* provides the enteric ecosystem with a diverse panel of saccharolytic functions, well suited to the array of gluco- and galacto-based saccharides that abound in the Western diet. On the other hand, the metagenomic functions assigned to *Treponema* are more predictive of a capacity to incorporate complex polysaccharides, such as those found in unrefined plant foods, which are consistently incorporated in the Hadza diet. Finally, unlike *Treponema*, the *Bifidobacterium* metagenome functions include genes that permit the establishment of microbe–host immunological cross-talk, suggesting recent co-evolutionary events between the human immune system and *Bifidobacterium* that are adaptive in the context of agricultural subsistence and sedentary societies.

## Introduction

The gut microbiota (GM) exerts a vital role in human physiology, being strategic for human nutrition, immune protection, and for the preservation of essential metabolic functions (Kau et al., 2011; Nicholson et al., 2012). This raises questions of whether, and to what degree, the human GM can provide the host with extra physiological and metabolic flexibility for the adaptation to different lifestyles and environments. Since 2010, several studies have been conducted with the specific aim to explore GM variation across human populations with different subsistence practices, lifestyles and geographical origin. These human GM surveys revealed the existence of robust GM compositional and functional subgroups, so far generally reflective of the variations in subsistence strategy: hunter-gatherer, rural agricultural, and urban industrial Western lifestyle (Yatsunenko et al., 2012; Schnorr et al., 2014; Martínez et al., 2015; Obregon-Tito et al., 2015; Rampelli et al., 2015).

The findings from this new and emerging field of research, which combines human microbiology ecology and anthropology, resulted in two main conclusions with important implications for both human evolutionary history and human health: first, humans co-evolved with symbiont microbial ecosystems, which have co-adapted along the trajectory of subsistence change across human evolutionary history, from hunter-gatherers to rural agricultural to the most recent development of completely industrialized societies (Quercia et al., 2014; Obregon-Tito et al., 2015); second, despite the considerable variation in rural and traditional life-ways, urban industrial populations stand apart as having a distinctly altered GM profile. Indeed, the GM of urban industrial populations seems to universally share certain compositional qualities, such as: (i) an overall compression of microbial diversity as measured by phylogeny and the number of unique taxa (Segata, 2015), (ii) the loss of the so-called microorganisms “old friends”, *Treponema* and *Succinivibrio* (Blaser and Falkow, 2009; Warinner et al., 2015), and (iii) the acquisition of *Bifidobacterium* as typical inhabitant of the adult gut (Schnorr et al., 2014; Martínez et al., 2015; Obregon-Tito et al., 2015).

In agreement with the multiple hit hypothesis (Sonnenburg and Sonnenburg, 2014), dietary changes, sanitization and antibiotic usage are all potential triggers that would explain the reduction of ecosystem diversity and the loss of co-adapted microbial communities commonly observed in the GM profile of urban industrial Western populations. Conversely, the factors that favored the correspondent acquisition of *Bifidobacterium* in the Western microbiome still remain to be defined. Showing a relative abundance that ranges from 3 to 10% of the total ecosystem, bifidobacteria are an abundant bacterial component in the GM of urban industrial, namely Westernized, adults, and also dominates the GM ecosystem of breast-fed infants, where this bacterial family accounts on average for 80% of the total community (Turroni et al., 2012; Bottacini et al., 2014; Milani et al., 2015b). The recent characterization of the bifidobacterial pangenome – 18,181 *Bifidobacterium* specific Cluster of Orthologous Genes (BifCOGs) from 47 sequenced type strains – revealed the saccharolytic functions of this microorganism, and indicated a strong adaptation to the human gut environment (Milani et al., 2015a). Furthermore, recent work has helped advance a possible hypothesis to explain the adoption of *Bifidobacterium* into the Western adult GM. Through comparisons of the GM of Hadza hunter-gatherers and urban industrial Italians, Schnorr et al. (2014) highlighted – for the first time to our knowledge – the substantial lack of *Bifidobacterium* from the GM of some traditional populations. The authors proposed that the lack of bifidobacteria in adult Hadza hunter-gatherers may be “a consequence of the post-weaning GM composition in the absence of agro-pastoral-derived foods”, while the continued consumption of dairy into adulthood is one of the possible vectors by which many Westernized populations maintain a relatively large bifidobacterial presence. Obregon-Tito et al. (2015) and Martínez et al. (2015) reached similar conclusions through comparative research on the GM of hunter-gatherers from Peru and rural highlanders from Papua New Guinea, respectively, and also found a low proportion of bifidobacteria relative to individuals from the USA. However, these comparative gut metagenome surveys do not specifically explore the impact of *Bifidobacterium* acquisition on the functional configuration of the GM of Western adults.

Taken together, these findings indicate a scenario of exchange, whereby through Westernization, human populations have lost long-standing commensal microorganisms, in particular *Treponema* and *Succinivibrio*, but have potentially compensated through the adult acquisition of bifidobacteria. In order to examine changes to the GM as a result of these community shifts, we investigate how the loss of *Treponema* and the acquisition of *Bifidobacterium* influenced the human gut metagenome profile. To this aim, we compare gut metagenome functions assigned to *Treponema* and *Bifidobacterium* retrieved from downloadable GM metagenomic data for both Hadza hunter-gatherers and urban Italians. Our findings reveal interesting functional gains in the Western microbiome corresponding to the post-weaning retention of *Bifidobacterium* as a symbiont microorganism, suggesting an opportunistic yet important role of this taxon in our recent history.

## Materials and Methods

### Sample Collection and Shotgun Sequencing

The Illumina shotgun sequences used in this study were previously generated (Rampelli et al., 2015), and are publically available at the National Center for Biotechnology Information – Sequence Read Archive (NCBI SRA; SRP056480, Bioproject ID PRJNA278393. Leipzig Ethik-kommission review board, reference number 164-12-21052012).

### *Bifidobacterium* and *Treponema* Species Identification within Italian and Hadza Metagenomes

In order to identify the *Bifidobacterium* and *Treponema* species in Italian and Hadza populations, respectively, the 16S rDNA sequences within the assembled metagenomes were taxonomically selected using the assign_taxonomy.py script of the Qiime pipeline (Caporaso et al., 2010), against the Greengenes database^1^. The assignment at species level was performed by blastn of the *Treponema* and *Bifidobacterium* 16S rDNA sequences against the entire NCBI nucleotide database, in particular the top hit results for each sequence were considered.

### Characterization of the CAZyme Repertoire Assigned to *Bifidobacterium* and *Treponema* in the Gut Metagenome

Reads from a total of 38 individual GM metagenomes, 27 Hadza and 11 Italians from Rampelli et al. (2015), were downloaded and included in this study. Reads were assembled into contigs using MetaVelvet (Namiki et al., 2012) with 350 bp as insert length. Predicted open reading frames (ORFs) were determined by FragGeneScan (Rho et al., 2010) on assembled contigs, using the “–w 0” option for the fragmented genomic sequences and the parameter “–t complete”. From the translated ORFs we detected the CAZymes-coding sequences using hmmscan tool from the HMMER software package (Eddy, 2011) and the dbCAN database (Yin et al., 2012). The outputs were processed by the script hmmscan-parser.sh^2^, selecting only the sequences that showed a minimum identity of 30% to the query sequences and an alignment length of at least 100 residues. In order to identify CAZymes derived from *Bifidobacterium* and *Treponema*, we retrieved the nucleotide sequences of the CAZymes detected with hmmscan from the FragGeneScan output, and we blasted them against the NCBI nucleotide database. Only the sequences that showed as best hit an assignment to *Bifidobacterium* for the Italian samples or *Treponema* for the Hadza samples were retained for further analysis. On the basis of the coverage of the contigs, we obtained information concerning the abundance of CAZymes. To compare the data among samples, we obtained a normalized CAZyme abundance by dividing the CAZyme coverages of every correspondent contig for the giga-bases of every correspondent sample. Heatmaps and graphs were generated in R using the packages made4 (Culhane et al., 2005) and stats^3^.

### Read-Mapping Approach for the Detection of *Bifidobacterium* and *Treponema* Functions Involved in the Adaptation to the Gut Environment

High quality reads for each sample were aligned to *Bifidobacterium-* or *Treponema*-assigned genes encoding bile acid adaptation, host interaction, and polysaccharide catabolism using bowtie2 and setting the alignment parameters to “–sensitive-local” (See Supplementary Table S1 for the list of genes used for this analysis). As reference for the alignment, two different databases containing orthologous genes from the NCBI genomes of the previously detected *Treponema* or *Bifidobacterium* species were created. Specifically, the databases contain genes for alpha-amylase, beta-galactosidase, mannanase, cellulase, pectinase, and xylanase. Furthermore, the databases were implemented with the sequences of the bile eﬄux pump, bile salt hydrolase, exopolysaccharide synthase, fimbrial subunit FimQ, sortase, galactosyl transferase, and undecaprenyl-phosphate phosphotransferase, since they were recently reported as genes that facilitate commensal-host cross-talk in *Bifidobacterium* (Gueimonde et al., 2009; Fanning et al., 2012; Turroni et al., 2013; Ferrario et al., 2016). In the event that the *Bifidobacterium* or *Treponema* NCBI genomes did not contain the above-mentioned genes, we supplemented the databases with genes belonging to the taxonomically closest annotated microorganism. The reads that aligned with a reference using bowtie2, were extracted and their taxonomy was further verified by blastn against the entire NCBI nucleotide database. Notably, in the case that the best hits of the blastn search were not assigned to *Bifidobacterium* or *Treponema*, we did not consider those reads for further analysis. The number of hits for each gene was normalized by the number of base pairs in the input file and in the correspondent reference in order to compare the results.

## Results

### Variation in the Gut Microbiome Carbohydrate-Degrading Repertoire as a Result of the Exclusive Presence of *Bifidobacterium* or *Treponema* in the Italian and Hadza Gut Microbial Ecosystems

By studying hunter-gatherers and rural populations, we previously depicted an approximation of the co-evolutional trajectory of the human GM structure in the recent times (Schnorr et al., 2014). In particular, we characterized the taxonomic and functional metabolic potential of the GM in the Hadza hunter-gatherers and urban Italian adults, reporting considerable differences between the two populations that map on to their respective life-ways (Schnorr et al., 2014; Rampelli et al., 2015). Among them, we observed a loss of *Treponema* and an acquisition of *Bifidobacterium* in the Italian GM. Considering the importance of *Bifidobacterium* as a health-promoting commensal of the GM in Western populations, it is important to understand how this beneficial role may have developed through the lens of co-evolution.

**FIGURE 1:**
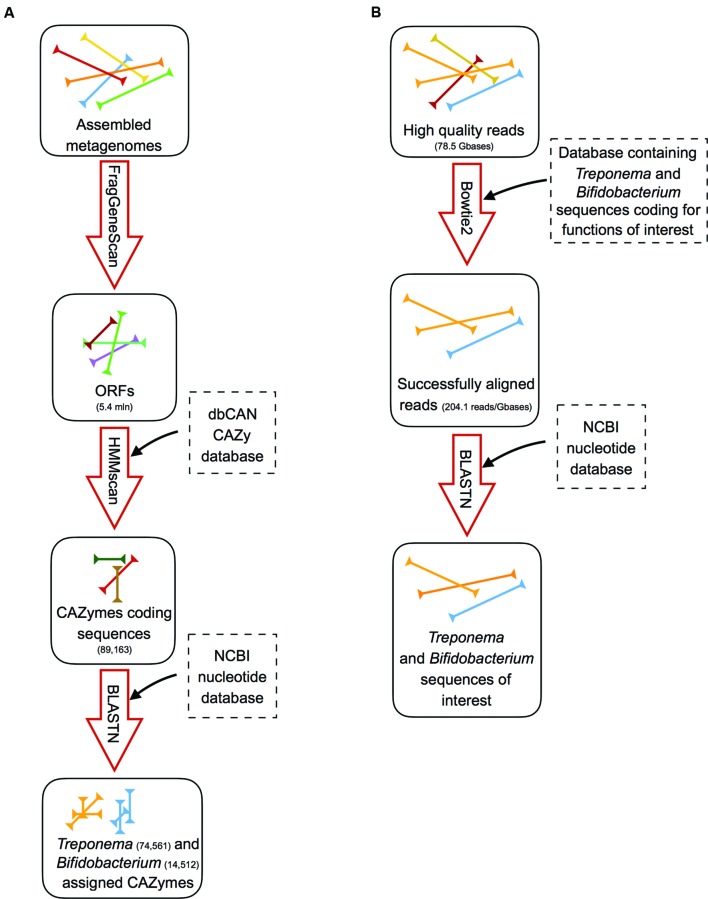
**Schematic representation of the analysis workflow. (A)** Pipeline for the identification and assignment of *Treponema* and *Bifidobacterium* CAZymes on assembled metagenomes: (i) ORFs detection using FragGeneScan; (ii) detection of the CAZyme-coding ORFs by using hmmscan against the dbCAN CAZy database; (iii) taxonomy assignment to CAZyme-coding sequences by blastn against the NCBI nucleotide database. **(B)** Pipeline for the identification of *Treponema* and *Bifidobacterium* sequences coding for functions involved in the adaptation to the gut environment: (i) alignment of high quality reads to databases containing the selected *Treponema* or *Bifidobacterium* functions using bowtie2; (ii) blasting of the successfully aligned reads against the NCBI nucleotide database to confirm the taxonomy.

**FIGURE 2:**
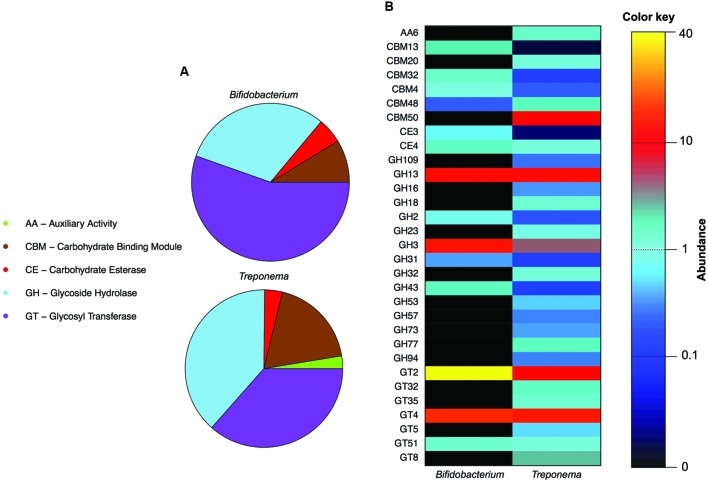
**Overview of *Bifidobacterium* and *Treponema* CAZyme repertoires in the Hadza and Italian samples. (A)** Normalized relative abundance of the CAZyme category levels for *Bifidobacterium* and *Treponema*: auxiliary activity (AA), carbohydrate binding module (CBM), carbohydrate esterase (CE), glycoside hydrolase (GH), and glycosyl transferase (GT) categories. **(B)** Comparison between the *Bifidobacterium* and *Treponema* CAZyme family profiles. The relative abundance of each family is indicated by the color key.

**FIGURE 3:**
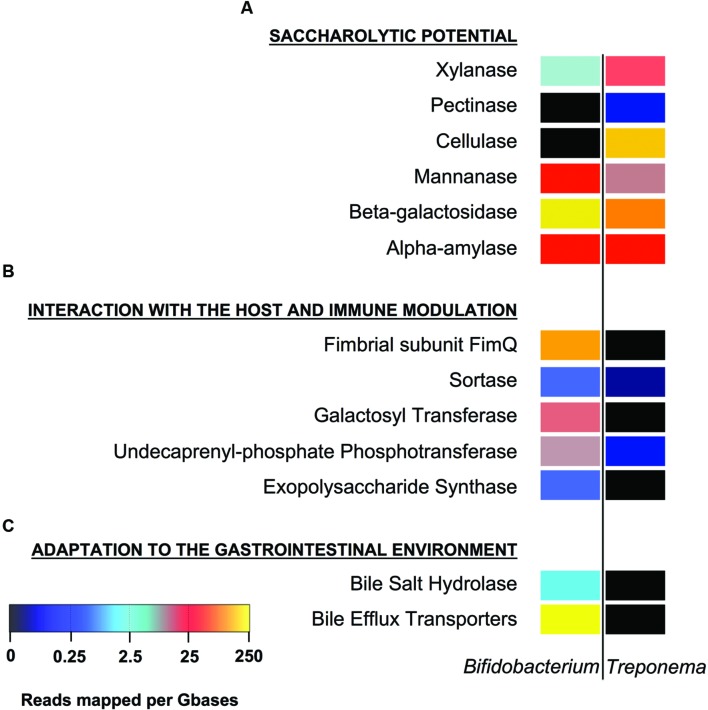
**Profile of *Bifidobacterium* and *Treponema* functions involved in the adaptation to the host environment**. Polysaccharide metabolism **(A)**; interaction with the host and immune modulation **(B)**; adaptation to the human gastrointestinal environment **(C)**. Color key represents reads mapped per giga-bases of the sample of origin. See Supplementary Table S1 for the list of reference genes used.

We first identified the diversity of the *Bifidobacterium* and *Treponema* species in urban Italian and Hadza GM by reconstructing the full 16S rDNA gene from assembled metagenomes. Italian samples contain sequences belonging to *Bifidobacterium faecale*, *Bifidobacterium pseudocatenulatum*, *Bifidobacterium adolescentis*, *Bifidobacterium coryneforme*, *Bifidobacterium bifidum*, *Bifidobacterium longum*, *Bifidobacterium angulatum*, and *Bifidobacterium dentium*. On the other hand, we detect the 16S rDNA sequences assigned to *Treponema porcinum*, *Treponema bryantii*, *Treponema succinifaciens*, *Treponema parvum*, and *Treponema berlinense* in the GM of the Hadza hunter-gatherers. However, we must acknowledge that these taxonomic assignments are limited by present whole genome for *Treponema* species, most of which have been characterized by work on human pathogens, rather than commensal members of the GM. Ongoing work with species-level variation should be crucial to resolving the disparities in our classification and resolution of these species and their functions in the future (Obregon-Tito et al., 2015). In order to compare the specific saccharolytic functions conferred by *Bifidobacterium* and *Treponema* in the Italian and Hadza microbiomes, we identified a total of 5.4 million of ORFs, of which 14,512 mapped to CAZymes for the Italian samples and 74,651 for the Hadza samples (**Figure 1A**). Notably, the Hadza metagenomes contain significantly more CAZymes per subject, in terms of ORFs assigned to CAZymes per million of reads, respect to the Italian metagenomes (mean ± SD, Hadza: 233 ± 86, Italians: 137 ± 78), as reported by Rampelli et al. (2015). We then profiled the saccharolytic repertoire of *Bifidobacterium* and *Treponema* in the Italian and Hadza GM as relative abundance at the CAZyme category level based on the coverage of taxonomically assigned contigs (**Figure 2A**). *Bifidobacterium* showed a higher presence of glycosyl transferase (GT) and carbohydrate esterase (CE), with respect to *Treponema* (GT relative abundance, rel. ab.: 54% for *Bifidobacterium* and 43% for *Treponema*; CE rel. ab.: 6% for *Bifidobacterium* and 5% for *Treponema*). On the other hand, *Treponema* were more enriched in glycoside hydrolase (GH) and carbohydrate binding module (CBM) (GH rel. ab.: 33% for *Treponema* and 31% for *Bifidobacterium*; CBM rel. ab.: 15% for *Treponema* and 8% for *Bifidobacterium*). At the CAZyme family level, we revealed the four families that constitute the core *Bifidobacterium* CAZyme repertoire: GH13 (GH family acting on substrates containing α-glycosidic linkages), GH3 (GH family that groups together exo-acting β-D-glucosidases, α-L-arabinofuranosidase, β-D-xylopyranosidase and *N*-acetyl-β-D-glucosaminidase), GT2 (GT family containing cellulose synthase, mannan synthase, and several monosaccharide-/oligosaccharide-transferases), and GT4 (GT family containing sucrose synthase, glucosyl transferase, and several phosphorylases). The sum of ORFs assigned to these four major families comprises 77% of the total detected CAZyme cohort. The relative abundance of the *Bifidobacterium* and *Treponema* CAZyme families detected in the Italian and Hadza samples reveals several differences in the potential saccharolytic functional contributions of these two microorganisms (**Figure 2B**). In particular, *Bifidobacterium* have a greater abundance of genes involved in the degradation of lactate, which is produced from pyruvate in the fermentation of simple sugar and commonly found in sour milk as well as in other lacto-fermented foods (family GH2). This finding also corresponds to the exclusive contribution of dairy carbohydrates (∼5% of total carbohydrates), in the Italian cohort diet (Supplementary Table S2). Emphasis on monosaccharide catabolism is evidenced by enrichment in gene families containing enzymes that metabolize mannose, xylose and arabinose (GH2, GH31, and GH43), which are highly represented in plant and fruit glycans. As a variety of genes were also found that are involved in the degradation of α- and β-glucans (GH3 and GH31), this illustrates an ability of *Bifidobacterium* to retrieve energy also from more complex carbohydrates that are commonly present in the cellulosic biomass of plant foods in the Italian diet: salads, fruits, nuts, cereals and their product derivatives. In addition, *Bifidobacterium* are also enriched in genes involved in the catabolism of sucrose (GH31), which is widely distributed in nature, but robustly manifest in the industrial food products that are consumed daily by most urban populations. Further evidence of these functions comes from detection of a higher abundance of CBM families for lactose, galactose and β-glucans (CBM4, CBM13, and CBM32) in *Bifidobacterium*, with respect to *Treponema*. In contrast, the CAZyme profile of *Treponema* within the Hadza metagenome is mainly devoted to degradation of glucans, galactans, and fructans (GH16, GH32, and GH53), which are sugar polymers that comprise hemicellulose (galactans) and inulin (fructans). The monosaccharide of galactans, galactose, is also expressed in mucilages and glycoproteins that derive from the human host, as well as a number of vegetable-derived carbohydrates. Both sugar polymers are largely implicit in difficult-to-digest plant polysaccharides that escape small intestine absorption and are instead fermented by the colonic microbiota. The Hadza diet is replete with such unrefined plant foods that contain indigestible polysaccharides such as berries, baobab fruit, and particularly tubers (Schnorr et al., 2015). *Treponema* are also enriched in two CAZyme α-amylase families (GH57 and GH77), which are unlike the typical α-amylase GH13 family because they have a conserved trans-glycosylating region (MacGregor et al., 2001). Finally, *Treponema* are better equipped to metabolize peptidoglycans due to a wide range of acetylglucosaminases and peptidoglycan lyases (GH23, GH73, and GH109). These activities were confirmed by the detection of high levels of CBM families for peptidoglycans and α-glucans (CBM50 and CBM48). To specifically investigate the effective differences in polysaccharide-degrading repertoires between *Bifidobacterium* and *Treponema* metagenome functions, we aligned the reads of Italian and Hadza samples to a custom database containing the sequences for alpha-amylase, beta-galactosidase, mannanase, cellulase, pectinase, and xylanase. The taxonomic specificity of the aligned reads was verified by blasting the sequences to the nucleotide database of NCBI (**Figure 1B**). Unlike *Treponema*, which appears to be functionally equipped to derive energy from a broad spectrum of polysaccharides through increased relative representation of pectinase, xylanase, and cellulase, the polysaccharide-degrading functions assigned to *Bifidobacterium* are enriched in genes coding for β-galactosidase and mannanase (**Figure 3**). Interestingly, both genera are configured to hydrolyze 1–4 α-glycosidic bonds (via α-amylase) to a similar degree. The catabolic configurations of *Bifidobacterium* and *Treponema* for polysaccharides are reflective of differences in dietary carbohydrates of Italians and Hadza: pasta, legumes, milk, and dairy products, versus berries, tubers, baobab, and honey, respectively, (Supplementary Table S3).

### Differences in the Gut Metagenome Functions Involved in Bacteria/Host Interaction and Gut Adaptation Corresponding to the Presence of *Bifidobacterium* or *Treponema* in the Italian and Hadza Gut Metagenome

We further investigated the presence of the genes involved in host interaction and immune modulation in the ORFs attributed to *Bifidobacterium* and *Treponema*. In this scenario, the sortase-dependent pili are considered key surface molecules in establishing bacterial adherence to the host epithelium and they have been proposed as potential mediator of mucosal immune response (Turroni et al., 2013). Another recognized marker of interaction with the host that affects the immune response is the surface-exopolysaccharide (EPS). Specifically, bacteria producing EPS failed to elicit a strong immune B-cell response compared to EPS-deficient strains (Fanning et al., 2012). Our analyses confirm that the enzymes involved in the production of EPS and pili, namely EPS synthase, undecaprenyl-phosphate phosphotransferase, galactosyl transferase, sortase, and fimbrial subunit FimQ, are typical of the *Bifidobacterium* ORFs detected in the Italian metagenome, while virtually absent in the *Treponema* ORFs retrieved from the Hadza GM ecosystem (**Figure 3**). Finally, we investigated the presence of genes involved in bile tolerance as mechanisms of bacterial adaptation to the human host. Bile salts are detergent-like compounds with strong antimicrobial activity (Begley et al., 2005), and intestinal bacteria have had to evolve strategies to tolerate physiological concentrations of bile salts to colonize the intestine (Gueimonde et al., 2009). Interestingly, two representative enzymes, which contribute to bile resistance and adaptation to gut environment, the bile-inducible eﬄux transporters and the bile salt hydrolase, are present in within the *Bifidobacterium* gut metagenome functions, but are not detected in *Treponema* ORFs (**Figure 3**).

## Discussion

In our work we explore how the apparent dichotomy between two instance-specific mutually exclusive gut inhabitants, *Bifidobacterium* and *Treponema*, reflects specific functional roles within the human GM ecosystem. We observe that the complete replacement of these microorganisms within the human gut in modern populations follows a curious pattern of lifestyle-associated differences, which stand at opposite ends of an entire subsistence spectrum, from hunting and gathering to post-industrial urban life. This may hint at a more ancient pattern of transition that occurred along more recent human evolutionary history as early settlements permitted the adoption of a fully agro-pastoral regime in place of mobile foraging. As seen in modern rural farmers, these earlier human groups may have harbored both communities of bifidobacteria and treponemes, suggesting an intermediary phase of co-habitation (De Filippo et al., 2010; Obregon-Tito et al., 2015). These patterns are instructive, and we can hypothesize that the stimulus for bifidobacterial acquisition and proliferation in the human GM followed much earlier changes to diet and lifestyle that occurred during the Neolithic transition. Specifically, these changes include permanent settlements, dense population structures, animal co-habitation, plant domestication, and shifts in the type and amount of carbohydrate-based foods (Childe, 1936; Binford, 1968).

While bifidobacteria are absent from the GM ecosystem of modern hunter-gatherers, and thus possibly acquired as a post-weaning symbionts among communities who adopted an agro-pastoral subsistence, *Treponema* has been lost from the human GM along the transition from small-scale rural communities to post-industrial urbanized society. This raises key questions such as ‘what are the functional gains and losses in the human GM corresponding to this process?’, and ‘does an adaptive nature of these changes exist?’ To begin answering these questions, we compared metagenome functions assigned to *Bifidobacterium* and *Treponema* in the gut metagenome samples from urban Italians and Hadza hunter-gatherers, respectively. In particular, we focused on functional features key in the context of the GM-human host mutualism, such as the metabolism of complex polysaccharides, which is essential to provide the host with short-chain fatty acids (SCFA), and the microbe–host interaction and immunomodulation processes.

As an initial approach, we described the *Bifidobacterium* and *Treponema* diversity in the urban Italian and Hadza GM. Eight different bifidobacterial species were detected in the Italian GM ecosystem, including the plant polysaccharides degrader *B. adolescentis*, and the milk fermenters *B. bifidum* and *B. longum*. Other bifidobacterial species detected in the Italian GM were *B. faecale*, *B. pseudocatenulatum*, *B. coryneforme*, *B. angulatum*, and *B. dentium*, highlighting considerable bifidobacterial diversity within the Italian GM ecosystem. Given our current limitation in resolving novel *Treponema* taxa, we were still able to identify five species: *T. porcinum*, *T. bryantii*, *T. succinifaciens*, *T. parvum*, and *T. berlinense*. These species were already described in association with the GM of Matses hunter-gatherers from Peru (Obregon-Tito et al., 2015).

According to our findings, *Bifidobacterium* and *Treponema* provide the host metagenome with a different configuration of CAZyme categories. Indeed, the *Bifidobacterium* genes encoding for CAZymes in the Italian gut metagenome are enriched in GT and CE, while the *Treponema* saccharolytic repertoire in the Hadza gut is principally devoted to GH and CBM functions, suggesting the higher propensity of the latter to act as a primary degrader of complex polysaccharides in the gut. Focusing on the CAZyme family level, we observed that *Bifidobacterium* provides the host metagenome with an extremely versatile panel of saccharolytic functions. Bifidobacterial CAZyme families enriched in the Italian samples range from the metabolism of simple sugars, such as lactate, mannose, xylose, and arabinose, to more complex carbohydrates from plant sources, such as α- and β-glucans, lactose, or galactose. These findings are in general agreement with the overall structure of the bifidobacterial glycobiome (Milani et al., 2015a). According to Milani et al. (2015a), when comparing the bifidobacterial GH repertoire with the sequenced members of the human GM, *Bifidobacterium* appears enriched in GH3 and GH43 families for the degradation of plant carbohydrates. These findings are in agreement with our observations that the *Bifidobacterium*-assigned saccharolytic functions emphasize substrate-specific specializations that are not observed among *Treponema*-assigned genes. Instead, the saccharolytic functions supplied by *Treponema* to the Hadza gut metagenome are mainly devoted to the degradation of indigestible polysaccharides, such as galactans, fructans, and glucans, that are present in unrefined and wild plant foods. Other functional peculiarities characteristic of *Treponema* include a completely unique suite of α-amylases and a greater capacity to bind and metabolize peptidoglycan. When we specifically explored gut metagenome differences in polysaccharide-degrading genes, we confirmed the overall greater metabolic polysaccharolytic potential for *Treponema* while *Bifidobacterium* was enriched in beta-galactosidase and mannase.

Interestingly, the specific glycobiomes conferred by *Bifidobacterium* and *Treponema* match the respective dietary habits of the two populations. Hadza consume a heavily plant-based diet, particularly in the rainy season, in which ∼70% of kcal are derived from fibrous wild plant foods and the 30% from wild game meat (Marlowe, 2010). Furthermore, Hadza entirely lack dairy in their diet. Correspondingly, the *Treponema*-assigned glycobiome in the Hadza is devoted to the metabolism of the vast array of refractory glucans, galactans, and fructans that are present in the wild Hadza plant foods. The Mediterranean diet, characteristic for the Italian cohort, is abundant in plant foods (salads, fruits, sauces), pasta, bread and olive oil with low to moderate inclusion of dairy, poultry, fish and read meat (Supplementary Table S3). Curiously, certain glycobiome functions conferred by bifidobacteria to the Italian GM – e.g., GH2, GH31, and GH43, as well as CBM4, CBM13, and CBM32 – are well suited to deal with dairy carbohydrates and some types of plant glycans, all of which are relatively abundant in the Italian diet.

When we looked for functions associated with host–microbe interactions, we found matches only among *Bifidobacterium*. Conversely, *Treponema* does not provide the Hadza metagenome with known functionalities for direct host interaction. The *Bifidobacterium* functional potential encoded within strains retrieved from the Italian samples included enzymes for the biosynthesis of pili and EPS structures. Both cell components are essential in the context of a mutualistic strategy that permits close interaction and initiation of a tolerated immunological dialog between the bacterium and host (Fanning et al., 2012). Finally, as a functional marker of the bacterial adaptation to the gut environment (Begley et al., 2005), we sought functions involved in bile salts tolerance. Interestingly, only bifidobacteria seem to possess bile-inducible eﬄux transporters and the bile salt hydrolase required for bile salt mitigation and detoxification. Even if biases in the assignment of *Treponema* functions as a result of the paucity of reference genomes cannot be excluded, our findings could indicate different ecological strategies for *Bifidobacterium* and *Treponema* in the human gut.

## Conclusion

Our findings suggest possible co-evolutionary implications for the loss of *Treponema* and the acquisition of *Bifidobacterium* as a stable component of the post-weaning GM ecosystem from post-industrial urban populations. Capable of heterogeneous saccharolytic metabolism, which ranges from complex plant polysaccharides to simpler sugars such as lactose and sucrose, *Bifidobacterium* are well suited to handle the degradative demands imposed by a typical Western diet. Conversely, the progressive loss of more challenging microbiota accessible carbohydrates in the Western diet (Sonnenburg et al., 2016), such as hemicellulose and inulin, would help partially explain the extinction of a more specialized fiber degrader such as *Treponema* from the Western GM ecosystem. Furthermore, unlike *Treponema*, *Bifidobacterium* evolved the capacity to establish an intense microbe–host connection, which may help support a continuous and abundant bifidobacterial presence in adults, allowing this commensal to outcompete other opportunistic, but functionally diverse, microbiota. The acquisition of *Bifidobacterium* as a stable component of the GM ecosystem in small-scale rural agriculturalists, reminiscent of early human farmers, and modern Westernized populations, may therefore engage the functionalities of the host immune system, providing new adaptive solutions in response to changing selective pressures during the restructuring of human diet and society.

## Author Contributions

SR and MC conceived the study with the participation of MS in experimental design. MS and SR performed the bioinformatics analysis. MS, SR, SLS, ST, and MC carried out data interpretation and wrote the paper. SQ, AC, EB, and PB participated to the discussion of the results and in revising the final draft of the paper. All authors read and approved the final manuscript.

## Conflict of Interest Statement

The authors declare that the research was conducted in the absence of any commercial or financial relationships that could be construed as a potential conflict of interest.

## Footnotes

1 http://greengenes.secondgenome.com/downloads

2 https://github.com/carden24/Bioinformatics_scripts/blob/master/hmmscan-parser.sh

3 https://stat.ethz.ch/R-manual/R-devel/library/stats/html/00Index.html
