# Supplementary material for: Variations in the Post-weaning Human Gut Metagenome Profile As Result of Bifidobacterium Acquisition in the Western Microbiome
Source: Front Microbiol. 2016 Jul 12;7:1058. doi: 10.3389/fmicb.2016.01058 (PMC4940381; doi:10.3389/fmicb.2016.01058)
Supplement: Supplementary file 2 [file Table_2.PDF]

Supplementary table 2. Percent contribution of food categories and corresponding nutrients to average daily Italian cohort diet (from Schnorr et al., 2014).

Food Category	Most common food items	Energy (kcal)	Fat (g)	Protein (g)	All carbohydrate (g)	Fiber (g)	Sugar (g)	Other carbohydrate (g)
**Bread, cereal, pasta, grain**		37.2%	25.3%	33.6%	48.3%	45.2%	16.0%	74.0%
	flatbread, brioche, brown bread							
	pasta: tortellini, lasagna, noodles							
	cereals, biscuits, crackers, pizza crust							
**Other starch**		3.7%	1.2%	2.3%	5.7%	6.3%	0.7%	8.5%
	beans, legumes							
	rice, potatoes, couscous							
**Meats, egg, meat broths**		10.7%	16.0%	31.2%	0.7%	0.4%	1.0%	0.5%
	pork: cured, steak, roast, salami							
	chicken: baked, roasted							
	beef: steak, roast, sausage,							
	fish: tuna, sole, cod, bass, flounder							
**Dairy**		13.0%	22.3%	19.9%	5.1%	0.5%	13.0%	0.7%
	milk, cream, yogurt							
	cheese: parmesan, mozzarella, sheep							
**Fruit, fruit juice**		8.8%	0.8%	2.4%	17.2%	19.9%	29.4%	5.6%
	banana, apple, pear, persimmon, citrus							
	orange juice, pear juice							
**Vegetables, sauces, herbs**		4.6%	3.1%	5.4%	6.1%	23.1%	4.9%	4.6%
	lettuce, carrot, onion							
	tomato sauce, mixed vegetables							
	boiled cabbage, squash							
**Desserts, sugary treats**		12.6%	14.6%	4.0%	14.6%	2.8%	33.3%	4.8%

	sugar, honey, cake, jam, soda							
	chocolate, pastries, ice cream							
**Oil, butter, fat**		4.4%	13.6%	0.0%	0.0%	0.0%	0.0%	0.0%
	olive oil, butter							
**Alcohol**		3.5%	0.0%	0.3%	1.4%	0.0%	0.3%	0.8%
	beer, wine							
**Nuts**		1.1%	3.1%	0.8%	0.2%	1.2%	0.1%	0.2%
	walnut, peanut							
**Other**		0.4%	0.1%	0.1%	0.6%	0.5%	1.3%	0.2%
	espresso, coffee, barley coffee, tea							
	vinegar, mustard, salt							
**Average daily amount**		**1852.3**	**66.3**	**75.0**	**236.5**	**20.0**	**84.5**	**120.6**

Percent contribution of each food group for energy (kcal), fat (g), protein (g), and carbohydrate (g). Carbohydrate is further broken down into types of carbohydrate with percent contribution by gram of the following: fiber, sugar, and other carbohydrate (oligosaccharides). An average daily intake for each category is reported in the last row.
