# Supplementary material for: Variations in the Post-weaning Human Gut Metagenome Profile As Result of Bifidobacterium Acquisition in the Western Microbiome
Source: Front Microbiol. 2016 Jul 12;7:1058. doi: 10.3389/fmicb.2016.01058 (PMC4940381; doi:10.3389/fmicb.2016.01058)
Supplement: Supplementary file 3 [file Table_3.PDF]

Supplementary table 3. Summary of Hadza foods and nutrition (from Schnorr et al., 2014).

					g 100g^{-1} dry wt.					kcal 100g^{-1} dry wt.
**Food type**	**Hadza name**	**English name**	**Species name**	**Moisture (%)**	**Edible portion**	**Carbohydrate**	**Protein**	**Fat**	**Fiber**	**Energy**
**Baobab**	N//obabe (pulp)**	Baobab	*Adansonia digitata*	4.7	na	46.6	2.5	0.7	45.1	203.0
	N//obabe (seed)**	Baobab	*Adansonia digitata*	4.8	na	11.2	36.3	29.3	14.1	454.0
**Berry**	Kisinubi	na	*Cordia sinensis*	73.0	na	68.8	12.6	1.8	11.6	342.0
	Undushibi**	na	*Cordia sinensis*	71.0	na	61.4	15.2	na	13.6	324.0
	Masakapi	na	*Cordia crenata*	69.0	na	62.5	12.7	1.9	17.8	318.0
	Hlukayebe	na	*Grewia villosa*	24.0	na	72.7	7.1	na	13.4	337.0
	Kongolubi	na	*Grewia bicolor*	26.0	na	66.1	12.0	2.0	13.2	330.0
	Pawe	na	*Sclerocarya birrea*	83.0	na	49.9	3.6	na	37.7	232.0
**Honey**	Ba'alako**	Honey (stinging)	*Apis mellifera*	15.1	na	89.1	3.3	7.2	na	434.0
	N//ateko**	Honey (stingless)	*Meliponinae sp.*	23.5	na	92.8	3.2	3.2	na	412.5
**Meat**	Gewedako**	Dik-dik	*Madoqua kirkii*	na	na	na	na	na	na	na
	Tsokwonako**	Giraffe	*Giraffa camelopardalis*	na	na	na	na	na	na	na
	Chacha**	Galago (lesser)	*Galago senegalensis*	na	na	na	na	na	na	na
	Ndonoko**	Galago (greater)	*Otolemur crassicaudatus*	na	na	na	na	na	na	na
	Tsunako**	Bee larvae	*Apis mellifera*	na	na	na	na	na	na	na
**Tuber**	//Ekwa hasa**	na	*Vigna frutescens*	68.8	39.4	60.8	7.2	0.8	28.2	282.3
	//Ekwa gadabi	na	*Vigna fructescens*	70.0	na	60.6	8.0	1.9	22.7	84.5
	Shumuko**	na	*Vatoraea pseudolablab*	90.1	na	50.8	3.3	0.6	35.2	225.0
	Do'aiko	na	*Vigna macrorhyncha*	85.0	na	58.6	10.4	0.1	22.4	292.0
	Matukwaiko	na	*Coccinea surantiaca*	86.5	na	69.5	12.4	0.0	13.3	337.0
	Mak'alitako**	na	*Eminia entennulifa*	71.7	56.9	26.4	4.1	1.1	17.1	110.1
	Panjuko**	na	*Ipomoea transvaalensis*	70.6	91.8	54.8	4.2	1.7	2.6	233.0
